# Microbial community structure elucidates performance of *Glyceria maxima* plant microbial fuel cell

**DOI:** 10.1007/s00253-012-3894-6

**Published:** 2012-02-25

**Authors:** Ruud A. Timmers, Michael Rothballer, David P. B. T. B. Strik, Marion Engel, Stephan Schulz, Michael Schloter, Anton Hartmann, Bert Hamelers, Cees Buisman

**Affiliations:** 1 Sub-department of Environmental Technology, Wageningen University, Bornseweilanden 9, 6708 WG Wageningen, the Netherlands; 2 Department Microbe–Plant Interactions, Helmholtz Zentrum München, German Research Center for Environmental Health, Ingolstädter Landstrasse 1, 85764 Neuherberg, Germany; 3 Department Terrestrial Ecogenetics, Helmholtz Zentrum München, German Research Center for Environmental Health, Ingolstädter Landstrasse 1, 85764 Neuherberg, Germany

**Keywords:** 454 amplicon sequencing, *Geobacter*, Microbial community, Plant microbial fuel cell, Renewable energy, Rhizosphere

## Abstract

The plant microbial fuel cell (PMFC) is a technology in which living plant roots provide electron donor, via rhizodeposition, to a mixed microbial community to generate electricity in a microbial fuel cell. Analysis and localisation of the microbial community is necessary for gaining insight into the competition for electron donor in a PMFC. This paper characterises the anode–rhizosphere bacterial community of a *Glyceria maxima* (reed mannagrass) PMFC. Electrochemically active bacteria (EAB) were located on the root surfaces, but they were more abundant colonising the graphite granular electrode. Anaerobic cellulolytic bacteria dominated the area where most of the EAB were found, indicating that the current was probably generated via the hydrolysis of cellulose. Due to the presence of oxygen and nitrate, short-chain fatty acid-utilising denitrifiers were the major competitors for the electron donor. Acetate-utilising methanogens played a minor role in the competition for electron donor, probably due to the availability of graphite granules as electron acceptors.

## Introduction

The plant microbial fuel cell (PMFC) is a new technology that can potentially provide renewable and sustainable energy. The PMFC transforms solar energy into electricity through the oxidation of organic compounds originating from photosynthesis (De Schamphelaire et al. 2008; Strik et al. 2008). Electricity generation in the PMFC is based on the loss of organic compounds by plant roots (rhizodeposition) (Pinton and Varanini 2007) and oxidation of these organic compounds by electrochemically active bacteria (EAB) (Potter 1911). In the PMFC, electrons, proton and carbon dioxide are produced by oxidation of organic compounds lost by plant roots in the anode. The electrons that are transferred to the anode electrode are consumed in the cathode compartment by typically reduction of oxygen to water (Strik et al. 2011).

Several bacterial species are known to produce current in microbial fuel cells (MFC): *Shewanella putrefaciens* using lactate, pyruvate and formate as electron donor (Kim et al. 1999; Park and Kim 2001); *Clostridium butyricum* and *Clostridium beijerinckii*, using glucose, starch, lactate and molasses (Niessen et al. 2004; Park et al. 2001); *Geobacter sulfurreducens*, using acetate and hydrogen (Bond and Lovley 2002); *Rhodoferax ferrireducens*, using glucose (Chaudhuri and Lovley 2003); *Geobacter metallireducens*, using acetate (Min et al. 2005) and *Rhodopseudomonas palustris*, using acetate, lactate, valerate, fumarate, ethanol, glycerol and yeast extract (Xing et al. 2008). *Alcaligenes faecalis*, *Enterococcus gallinarum* and *Pseudomonas aeruginosa* are known to generate electricity by producing their own mediators, using glucose as an electron donor (Rabaey et al. 2004). To date, *Enterobacter cloacae* is the only pure culture that is known to generate electricity directly from more complex electron donors, such as cellulose (Rezaei et al. 2009).

Rhizodeposition is the loss and release of organic and inorganic compounds by plant roots into the rhizosphere (the soil volume affected by the presence of plant roots). Rhizodeposition provides a carbon and energy source, thus stimulating the development of bacterial communities in the rhizosphere. In anaerobic environments, rhizodeposition can also provide oxygen as an electron acceptor and may stimulate the development of facultative anaerobic bacterial communities (Hartmann et al. 2009). In addition to prokaryotes (bacteria and archaea), eukaryotes (such as fungi, protozoa, nematodes and meso- and macro-fauna) are also found in the rhizosphere (Phillips et al. 2003).

The genera *Geobacter* and *Desulfobulbus* have been found in PMFCs (De Schamphelaire et al. 2010). As both genera contain electrochemically active species that are enriched at the anode of MFCs (Holmes et al. 2004; Jung and Regan 2007), it can be assumed that EAB may have been present in the PMFC. However, no studies have yet confirmed the presence of active EAB in PMFCs. The presence of active EAB is required to prove their role in electricity production by PMFCs.

In a PMFC, the plant roots are located at the anode of the MFC, where rhizodeposition provides EAB with electron donor. Together the MFC anode and the rhizosphere make up a complex system. In this complex system, it is likely that different microorganisms compete for electron donor. Competition for electron donor among the different microorganisms results in a decrease of electron donor available for EAB and thus lower current generation. Analysis and localisation of the microbial community is needed to gain insight into the competition for electron donor in a PMFC.

In this study, we determined the microbial communities present in a PMFC rhizosphere and localised the active EAB in the rhizosphere and on the electrode of the PMFC. Additionally, we investigated the electron donor and electron pathways in the PMFC. We used 454 technology to sequence 16S rRNA gene amplicon libraries from bacteria and archaea. To prove the relevance of dominant pylogenetic groups and to localise those bacteria and archaea, fluorescent in situ hybridisation (FISH) in combination with confocal laser scanning microscopy (CLSM) was performed in samples of high-current-generating and low-current-generating *Glyceria maxima* (reed mannagrass) PMFC.

## Materials and methods

### Preparation of PMFC

**Fig. 1:**
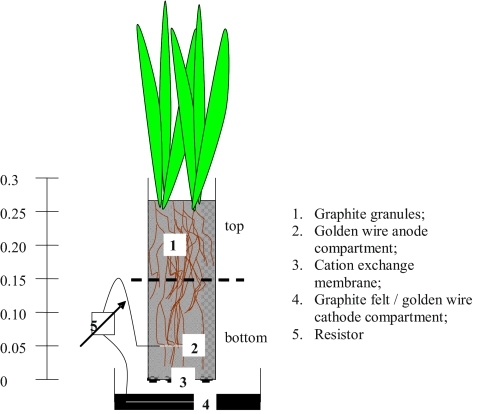
Schematic representation of the PMFC setup and location of the samples

The anode compartment of the PMFC consisted of 165 g of graphite granules with diameters between 1 and 2 mm (le Carbone, Wemmel, Belgium). The graphite granules were placed in a glass cylinder (Schott, Duran) with a diameter of 0.035 m and a height of 0.30 m. To separate the anode from the cathode, a cation exchange membrane (fumasep®, FKB, Fumatech GmbH, St. Ingbert, Germany) was fixed at the bottom of the glass cylinder. The anode was placed in the cathode, which consisted of a PVC beaker with a diameter of 0.11 m and a height 0.04 m that contained a 6-mm-thick graphite felt (Coidan Graphite Products LTD, York, UK). Figure 1 shows a schematic representation of the used setup.

To close the electrical circuit, two 4-cm golden wires glued to Teflon coated copper wire were connected over a resistance of 900 Ω. To create contact between the anode and the cathode, one of the gold wires was placed in between the graphite granules and the other was woven through the graphite felt.

### Preparation of graphite granules

Eight setups were operated without plants to develop electrochemically active biofilms on the graphite granules. The anode was inoculated with 5 ml of anolyte from an MFC running on acetate (Dekker et al. 2009), and the anolyte was extracted with a syringe. The setups were operated in batch mode. As nutrient solution 1/2 Hoagland Medium (Taiz and Zeiger 2006) buffered with an 8-mM potassium phosphate buffer solution (pH 6.8) was used. The electron donor was supplied by adding 10 mM potassium acetate to the nutrient solution of setups 1 through 4 and 10 mM glucose to the nutrient solution of 5 through 8. After 40 days, the acetate-fed setups generated a current density of 0.248 ± 0.004 A m^−2^, and the glucose fed setups generated a current density of 0.151 ± 0.02 A m^−2^. Current densities are expressed per square meter of cross-sectional area of the PMFC (0.00096 m^2^) throughout the document.

Before the graphite granules were used for the six PMFCs, they were thoroughly mixed to ensure the presence of microorganisms able to generate electricity from acetate as well as glucose. After the mixing, the graphite granules were rinsed with tap water to remove a large part of the residual electron donor. One stem of *G. maxima* (D’n Bart Waterplanten, Handel, the Netherlands) with a weight between 4.4 and 7.8 g was planted in each PMFC.

### Operation of PMFCs

The medium used to feed the PMFCs was 1/2 Hoagland buffered with 0.008 mol l^−1^ potassium phosphate buffer solution as described by Timmers et al. (2010) (pH 6.8, conductivity between 1.5 and 1.7 mS m^−1^). The medium was fed through a sample point located 0.07 m above the graphite granules. The applied flow rate was 0.17 ml s^−1^ throughout the experimental period. The feeding frequency of the PMFC with buffered Hoagland medium was 5 min every 12 h.

Both MFC and PMFC setups were placed in a climate control cabinet (Microclima 1750 Snijders, Tilburg, the Netherlands). In this manner, environmental conditions were fixed at illumination period of 14 h day^−1^, average light density in the photo active region of 596 ± 161 μmol m^−2^ s^−1^, temperature of 25°C and humidity of 75%.

### Sample selection, preparation and DNA extraction

Samples for analysis were taken from PMFC generating the highest current (PMFC6) and the PMFC generating the lowest current (PMFC3). Both PMFCs were dismantled 225 days after the start of the experiment. The anode compartments of both PMFCs were divided into bottom (0 to 0.15 m) and top (0.15 to 0.3) sections. Figure 1 shows a schematic representation of the PMFC and the sampling points. The root samples were taken from all of the sampling points in both PMFCs and stored at −80°C. DNA was extracted from a total of 500 mg of root material per sample following the protocol of Griffiths et al. (2000) and using a BIO101 lysing matrix (MP Biomedicals, Illkirch, France) and a final purification step with the AllPrep Mini Kit by Qiagen (Munich, Germany). The amount of DNA in solution was quantified using a bioanalyser from Agilent (Böblingen, Germany).

### PCR, 454 sequencing and data processing

If not otherwise mentioned, all the steps were performed according to the Roche 454 sequencing protocol for amplicons. To generate the amplicon library for bacteria and archaea, specific primers were selected according to two criteria: (a) the fragment should span 600 bases of the 16S rRNA gene to receive sufficient phylogenetic information and (b) the primers should bind to as many bacterial/archaeal sequences as possible without detecting non-target groups. To verify these criteria, the ARB probe match tool was used with the latest SILVA database (containing over 400,000 sequences), resulting in the following bacterial 16S primers: 926-F (5′-AAACTYAAAKGAATTGACGG-3′, *Escherichia coli* position 907–926 (Lane 1991)) and 630-R (5′-CAKAAAGGAGGTGATCC-3′, *E. coli* position 1528–1544 (Juretschko et al. 1998)). The archaeal primers were rSAf(i) (5′-CCTAYGGGGCGCAGCAG-3′, *E. coli* position 341–357 (Nicol et al. 2003)) and 958r (5′-YCCGGCGTTGAMTCCAATT-3′, *E. coli* position 940–958 (Bano et al. 2004)). These primers were extended as amplicon fusion primers with respective A and B adapters, key sequence and multiplex identifiers (MID) as recommended and tested in initial PCR reactions to determine their optimal annealing temperatures (50°C, 54°C, 58°C), cycle numbers (20, 22, 25, 30) and amounts of template DNA (50, 100, 200 ng). The conditions under which a sufficient amount (approximately 10^12^ molecules) of specific amplicons of the right size was generated using the lowest number of cycles in all the samples were determined (50°C, 22 cycles, 50 ng) for bacterial 16S rRNA genes. For archaeal 16S rRNA genes, 30 cycles and the addition of 8% DMSO were necessary to obtain sufficient PCR product for archaea. These conditions were then used to produce four amplicon libraries each (top and middle/bottom part of the high- and low-current-producing PMFC). The PCR products were purified with AMPure Beads (Agencourt, Beckman Coulter, Krefeld, Germany) and pooled in equimolar amounts.

Emulsion PCR, emulsion breaking of DNA-enriched beads and sequencing run of the amplicon pools were performed on a second-generation pyrosequencer (454 GS FLX Titanium, Roche) using titanium reagents and titanium procedures as recommended by the developer following protocols for bidirectional amplicon sequencing. Quality filtering of the pyrosequencing reads was performed using the automatic amplicon pipeline of the GS Run Processor (Roche) to remove failed and low-quality reads from raw data and to remove adaptor sequences.

Newbler assembler v 2.3 (Roche) was used to batch sequences based on MID-identifiers and to combine corresponding sequences derived from forward and reverse reads with a similarity of 99% and an overlap of 400 bases for the bacterial sequences. Due to the low sequence diversity, a similarity value of 98% and an overlap of 200 bases were sufficient for archaeal sequences. The consensus sequences were inspected for chimera with the help of the Bellerophon software (http://foo.maths.uq.edu.au/_huber/bellerophon.pl) and putative chimera were removed from the dataset.

The alignment and phylogenetic analyses were performed using the ARB 5.1 software package (Strunk and Ludwig 1997). After phylogenetic allocation of the sequences down to the family or genus level, the sequences belonging to different phylogenetic groups were added together and depicted as a percentage of the total sequencing reads obtained from each sample. Phyla represented by less than 50 sequencing reads in all four libraries totalled were not included. The assembled unique sequences with their phylogenetic allocation were deposited under the numbers JF731380 and JF732737 in GenBank.

### Quantitative real-time PCR of archaeal and bacterial 16S rRNA genes

For Sybr®Green-based quantitative real-time PCR (qRT-PCR), the same primers as for the 454 sequencing were used, but without the 454 specific adaptor, the key and the MID sequences. For standard generation, PCRs using these specific primers were performed with the same conditions as for the amplicon library amplification. The resulting products were cloned into a pSC-A-Amp/Kan Vector using the StrataCloneTM PCR Cloning Kit (Agilent, Palo Alto, CA, USA) after manufacturer’s instructions. In each case, eight resulting clones were picked, the inserts sequenced and subsequently allocated by the ARB software package. Sequences either related to the genus *Methanobacterium* (archaea) or to the genus *Clostridium* (bacteria) were chosen as standards, as both sequences represented genera commonly found in a large proportion in all four amplicon libraries.

Subsequently, abundances of archaeal and bacterial 16S rRNA genes were measured on the ABI Prism 3300 system (Applied Biosystems, Foster City, CA, USA) under comparable conditions (10 min 95°C, 40 cycles of 20 s 95°C, 1 min 50°C and 30 s 72°C) in triplicates. Signal acquisition was done at 78°C to overcome bias due to primer dimers. PCR reaction mixtures (25 μl volume) contained 2 mM MgCl_2_, 0.1 μM of respective primers (forward and reverse), 1× Power Sybr®Green (Applied Biosystems, Foster City, CA, USA) and 4 ng of template DNA. Curves for both standards were linear (*r* ^2^ > 0.99) over five orders of magnitude, and amplification efficiencies were comparable at 87%.

### Fluorescent in situ hybridisation and confocal laser scanning microscopy

Fixed bacterial samples were stained for 10 min in the dark with the DNA binding dye Syto Orange 80 (Molecular Probes, Invitrogen, Carlsbad, CA, USA) at a concentration of 5 μM in 10 mM Tris, 1 mM EDTA (pH 8.0) and subsequently washed twice with ultrapure water. For the FISH, the washed root samples were fixed for 2 h at room temperature (Amann et al. 1990) with either 50% ethanol in ultrapure water for Gram-positive bacteria or 4% paraformaldehyde for Gram-negative bacteria. Hybridisation with fluorochrome (Cy3, Cy5)-labelled oligonucleotide probes was carried out following the protocols described by Manz et al. (1992) and Amann et al. (1992). For the microscopic observations, the FISH stained root pieces (ca. 5–10 mm long) were embedded in Citifluor (Citifluor Ltd., Canterbury, UK) and observed by CLSM without further cutting.

rRNA-targeted oligonucleotide probes were synthesised and labelled by Sigma-Aldrich (Steinheim, Germany). EUB338-I (Amann et al. 1990) and EUB338-II and EUB338-III (Daims et al. 1999) were used in an equimolar mixture at different formamide concentrations. To detect the genus *Geobacter*, an equimolar mixture of the 16S rRNA targeted probes Geo1A and Geo1B was used at a 35% formamide concentration. Geo1A is specific for *G. sulfurreducens*, *Geobacter hydrogenophilus*, *Geobacter grbiciae* and *G. metallireducens*; Geo1B detects most other *Geobacter* species (Demaneche et al. 2008). To detect the *Ruminococcaceae* group members *Ruminococcus bromii*, *Clostridium sporosphaeroides* and *Clostridium leptum*, the Rbro730 probe (Harmsen et al. 2002) was applied in the presence of 20% formamide. To detect most members of *Clostridium* clusters I and II, the Chis150 probe (Collins et al. 1994; Franks et al. 1998) was used with 35% formamide.

The fluorescently labelled cells were detected using a Zeiss LSM510 CLSM. An argon ion laser supplied 488-nm illumination to excite the fluorescein, and two helium-neon lasers provided 543 nm for Cy3 and 633 nm for Cy5. For each hybridisation probe, an EUB338 mix labelled with Cy5 (blue) was combined with another group-specific probe labelled with Cy3 (red). The binding of both probes resulted in a magenta staining of the target cells. The green channel (fluorescein) served as a negative control that showed only autofluorescence by the root or other particles. Syto Orange was detected by excitation with the 488- and 543-nm laser wavelengths, resulting in a yellow/orange fluorescence signal in the target cells; the negative control was the blue channel (excitation at 633 nm).

The optical sectioning of the root was achieved by moving the focus position deeper into the sample in 1 μm steps, which produced z-stacks of individual pictures from the same *xy*-area with a penetration depth of up to 60 μm. The resulting set of pictures was displayed as an orthogonal view using the LSM510 software package that was provided by Zeiss.

## Results

### Energetic performance of PMFCs

**Fig. 2:**
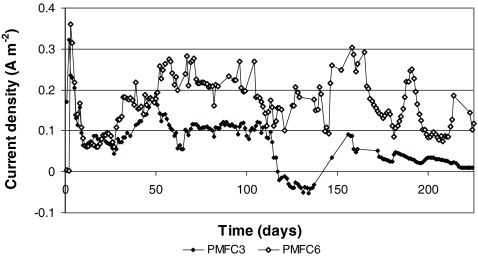
Current density versus time of the low-current-density PMFC (PMFC3) and high-current-density PMFC (PMFC6)

The daily average current density was 0.067 ± 0.058 A m^−2^ for the low-current PMFC and 0.164 ± 0.065 A m^−2^ for the high-current PMFC (Fig. 2). The daily average current density was substantially higher than the 0.009 A m^−2^ reported for freshwater sediment microbial fuel cells (Holmes et al. 2004). Furthermore, the daily average current density was in the range of average current densities reported in literature for PMFCs (De Schamphelaire et al. 2008; Kaku et al. 2008; Strik et al. 2008; Timmers et al. 2010; Helder et al. 2010). An examination of the standard deviations revealed that both PMFCs had relatively large fluctuations in current density over time. The maximum power density, with an external resistance of 900 Ω, was 32 mW m^−2^ for the low-current PMFC and 80 mW m^−2^ for the high-current PMFC. The high-current PMFC produced a total of 522 J, and the low-current PMFC produced a total of 124 J.

### Differences in the 454 sequencing of bacterial and archaeal 16S rRNA gene fragments between PMFC

A bidirectional sequencing approach of the four pooled bacterial 16S rRNA gene amplicon libraries (top section low-current PMFC, middle/bottom section low-current PMFC, top section high-current PMFC, middle/bottom section high-current PMFC) resulted in a total of 140,000 reads with 114,000 key pass reads and 46,000 reads that passed all of the internal quality filters of the 454 software. An average read length of 517 bases and almost no reads shorter than 500 bases were obtained. The sequencing of the bacterial amplicon libraries was repeated once for verification of the results with slightly lower output and read lengths but no major differences in bacterial abundance.

**Fig. 3:**
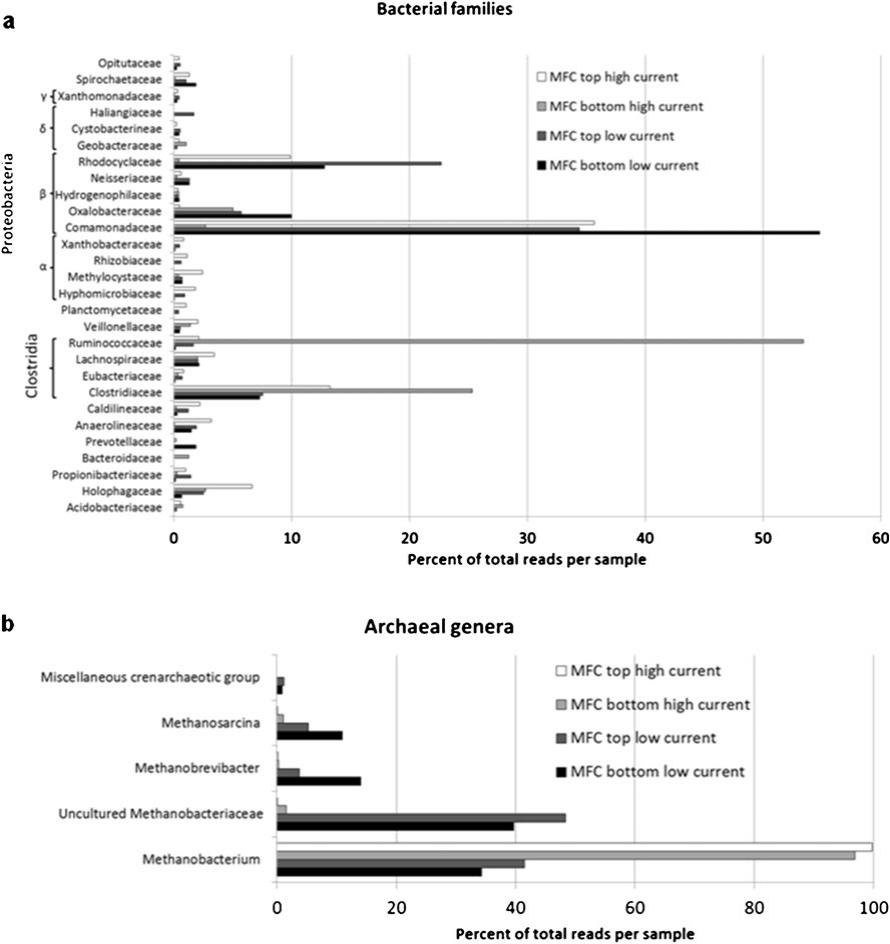
454 sequencing reads in percent of total reads per sample phylogenetically allocated to different families/genera by the ARB software package. Phyla represented by less than 50 sequencing reads in all four libraries totalled are not included. **a** Bacteria. **b** Archaea

The assembly resulted in 495 unique sequences for bacteria and 863 for archaea, which were then phylogenetically allocated by the ARB software package. Figure 3a shows the bacterial classes and families found in both PMFCs. In the bottom section of the high-current PMFC, the major portion (54%) of the bacterial 16S rDNA belonged to the *Ruminococcaceae* family, with considerable amounts (25%) belonging to the *Clostridiaceae* family. Within the *Ruminococcaceae* family, almost all the sequences clustered around *C. sporosphaeroides*. Although unambiguous species-level assignment is difficult using the 16S rDNA fragment lengths of 600 bp that were obtained, this analysis enabled the selection of suitable FISH probes for verification of the sequencing results.

In the top section of the high-current PMFC, the major portion (36%) of the bacterial 16S belonged to *Comamonadaceae*, and considerable amounts could be assigned to the *Rhodocyclaceae* (10%) and *Clostridiaceae* families (13%). The bottom and top sections of the low-current PMFC were dominated (54% and 35%, respectively) by members of the *Comamonadaceae* family, but also representatives of the *Rhodocyclaceae* family were frequently detected (13% and 23%).

Figure 3b shows the archaeal genera found in both PMFCs. In the high-current PMFC, the genus *Methanobacterium* made up over 95% of the archaeal community. In the low-current PMFC, the archaeal community was more diverse and mainly consisted of the genus *Methanobacterium* and uncultured *Methanobacteriaceae*. Furthermore, *Methanosarcina* was found to account for 5% of the archaeal community in the top section and up to 10% in the bottom section of the low-current PMFC.

The quantification of archaeal and bacterial 16S rRNA genes revealed great differences in the abundances of about two to three orders of magnitude. Gene copy numbers of bacterial 16S rRNA genes per nanogram extracted DNA were (top section, low current; middle/bottom section, low current; top section, high current; middle/bottom section, high current) 0.9, 2.4, 0.7 and 1.2 × 10^6^, respectively. In comparison, gene copy numbers of archaeal 16S rRNA were 5.6, 4.9, 1.4 and 0.8 × 10^3^, respectively. While the abundances of bacteria differed by a maximum of factor 2, when comparing the same sections of the high and the low-current PMFCs, there were four to six times more archaeal 16S genes in the low-current than in the high-current PMFC.

### Localisation of characteristic PMFC bacteria by FISH and CLSM

A biofilm was detected, particularly in roots from the upper sections of both PMFCs, where the film completely surrounded the root and was 5 to 10 μm thick. No visual differences in the structure or amount of the biofilms were observed between the PMFCs. For the low-current PMFC, however, fungal hyphae were clearly detected in the biofilms of all observed samples.

FISH was used to localise *Geobacter*, *Clostridiaceae* and *Ruminococcaceae* species that were previously detected by 454 sequencing of the 16S rRNA gene. The *Geobacter* probe set enabled detection of fluorescent *Geobacter* cells, which appeared more frequently on the graphite granules than on the root surfaces. With probe Geo1A, specific only for *G. sulfurreducens*, *G. metallireducens*, *G. grbiciae* and *G. hydrogenophilus*, positive signals were identified on both roots and granules from both PMFCs. Furthermore, the positive signal was more frequently observed on samples from the bottom layer of the high-current PMFC. A *Clostridia* cluster I and II specific probe yielded very few positive signals, and no clear differences were observed between the individual samples. With probe Rbro730, specific for the three *Ruminococcaceae* species *R. bromii*, *C. sporosphaeroides* and *C. leptum*, small numbers of cells were positively identified on the root surfaces, but fluorescent signals were detected to a much greater extent in the outer cortex layers of roots of the high-current PMFC.

## Discussion

### The presence of electrochemically active bacteria in the PMFCs

**Fig. 4:**
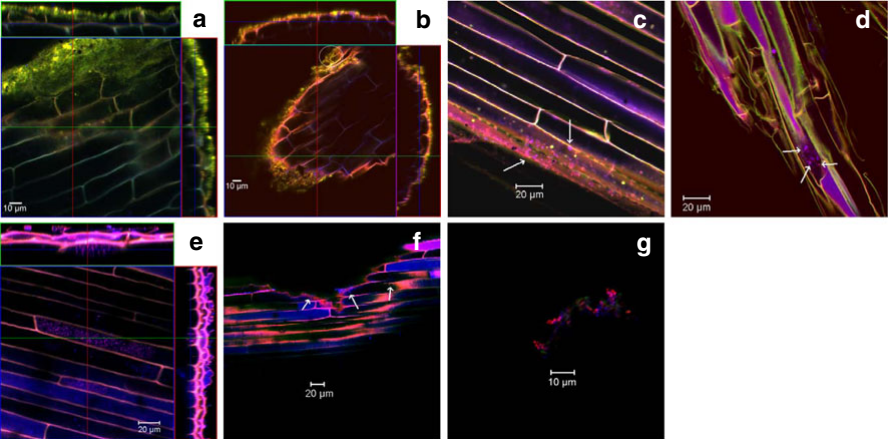
CLSM images of FISH or SYTO orange stained roots from the two PMFCs. All FISH staining was performed with probe Eub338Mix labeled in Cy5 (*blue*) in combination with a group specific probe labelled in Cy3 (*red*). Target cells appear in *magenta* (combination of *red* and *blue*). If three dimensional z-stacks were prepared, pictures are shown in orthogonal views. The top view, framed in *blue*, gives one picture from the middle of this z-stack. The *red* and *green lines* represent vertical optical cuts through the stack, which result in the side view images framed in *red* and *green*, respectively. In these side views, the *blue line* marks the vertical position, where the top view image is located within the z-stack. **a** Syto *orange* stained biofilm on a root from high-current-producing PMFC, *upper part*. **b** Syto *orange* stained biofilm on a root from low-current-producing PMFC, *upper part*; fungal hyphae are marked by *white circle*. **c** FISH stained root with probe set Geo1A and Geo1B (*Geobacter* genus) from high-current PMFC, *bottom part* (indicated by *white arrows*). **d** Root from same sample as 4C; Geo1A (detecting only *G. sulfurreducens*, *G. metallireducens*, *G. grbiciae* or *G. hydrogenophilus*, indicated by *white arrows*). **e** FISH stained root with probe Rbro730 (*C. sporosphaeroides*, *C. leptum*, *R. bromii*) from high-current PMFC, *middle part*. **f** FISH staining with probe set Geo1A and Geo1B (*Geobacter* genus) of a graphite granule of high-current PMFC (not visible); small cluster of *Geobacter* is detected on the surface of the graphite granules (indicated by *white arrows*). **g** CLSM picture of a FISH stained root from high-current PMFC, *bottom part*; probe Chis150 (*Clostridiaceae*)

The current generation was likely due to the presence of the active *G. sulfurreducens* and *G. metallireducens* that were detected in both the PMFCs (Fig. 4c). Pure cultures of both *G. sulfurreducens* and *G. metallireducens* are known to generate electricity in an MFC (Bond and Lovley 2002; Min et al. 2005). In particular, *G. sulfurreducens* has been shown to colonise the anodes of MFCs in structured biofilms (Holmes et al. 2004; Jung and Regan 2007; Reguera et al. 2006). The same probe also detected active *G. grbiciae* and *G. hydrogenophilus*. These species have not been shown to be capable of generating electricity in an MFC, but they are able to reduce Fe(III) (Lovley et al. 2000), a property that is also exhibited by EAB such as *S. putrefaciens*, *G. sulfurreducens*, *G. metallireducens* (Coates et al. 2001) and *R. ferrireducens* (Finneran et al. 2003).

In the bottom section of the high-current PMFC, *G. sulfurreducens* or one of its close relatives (*G. metallireducens*, *G. grbiciae* and *G. hydrogenophilus*) were observed frequently on the graphite granules. The roots in the bottom section of the high-current PMFC were also colonised, although less frequently than the graphite granules (Fig. 4c, f). In the low-current PMFC, *G. sulfurreducens* or one of its close relatives (*G. metallireducens*, *G. grbiciae* and *G. hydrogenophilus*) were observed less frequently in the top section, and no signal at all was detected in the bottom section.

The difference in the presence of the genus *Geobacter* between the PMFC types was supported by the differences in the presence of the *Geobacteraceae* family to which the *Geobacter* genus belongs (Fig. 4a). An average of 0.74% of the obtained 16S rRNA gene fragment sequences belonged to *Geobacteraceae* in the high-current PMFC, while an average of only 0.13% belonged to *Geobacteraceae* in the low-current PMFC.

### Anaerobic *Ruminococcaceae* were predominant in the bottom section of the high-current PMFC

*Ruminococcaceae* accounted for over half of the bacterial rhizosphere sequences in the bottom section of the high-current PMFC. The members of this family have not been reported to be electrochemically active, but they are known to hydrolyse cellulose into hydrogen and low molecular weight organic compounds. In MFCs, the hydrolysis of cellulose generates electron donors that are suitable for *G. sulfurreducens* (Maki et al. 2009; Ren et al. 2007).

Based on their cellulolytic activity, the *Ruminococcaceae* species probably played an important role in electricity generation in the PMFC. They probably hydrolysed the cellulose, originating from dead roots, into suitable electron donors for EAB. Typically, 35–50% of dry-plant weight consists of cellulose (Lynd et al. 1999), which stresses the importance of cellulolytic activity in generating a suitable electron donor for EAB.

The main species of the *Ruminococcaceae* family was probably *C. sporosphaeroides*, which is able to ferment glutamate to ammonia, CO_2_, acetate, butyrate and hydrogen via the hydroxyglutamate pathway (Hsiao et al. 2009). Furthermore, this species has been used to improve the hydrolysis rates of cellulose and hemicelluloses in a biomass fermenter (patent no. WO/2010/072219).

*C. sporosphaeroides* was mainly found in the root cells of the outer root cortex in the high-current PMFC (see Fig. 4e). Due to the large surface area of the outer root cortex, the presence of *C. sporosphaeroides* there probably resulted in a high rate of hydrolysis (Lynd et al. 2002). It could not be determined whether *C. sporosphaeroides* acted as a moderate pathogen and actively killed root cells or was simply colonising dead plant material. *E. cloacae*, able to generate current from complex electron donors as a pure culture, was not detected in the PMFCs (Rezaei et al. 2009). This is another indication that current was likely generated via the hydrolysis of complex electron donors into suitable electron donor for *G. sulfurreducens*. The importance of the hydrolysis of cellulose for electricity generation is supported by the results of Ren et al. (2007). They demonstrated that a binary culture of *Clostridium cellulolyticum* and *G. sulfurreducens* (with carboxymethyl cellulose and MN301 cellulose as electron donors) produced current, while single cultures of these bacteria did not.

The lack of dominance by obligate anaerobes in the top section of the high-current PMFC and in the top and bottom sections of the low-current PMFC indicates the presence of oxygen. Therefore, hydrolysis in the low-current PMFC was likely performed by fungi and/or facultative anaerobic cellulolytic bacteria, such as the *Rhodocyclaceae* family (Schellenberger et al. 2010). In contrast, the bottom section of the high-current PMFC was dominated by the obligate anaerobe *Ruminococcaceae*, indicating that cellulose hydrolysis was predominantly carried out by members of this family.

### Short-chain fatty acid-utilising denitrifiers are major competitors for electron donor

Except for the bottom section of the high-current MFC, *Rhodocyclaceae* and *Comamonadaceae* were the dominant families observed on the PMFCs. *Rhodocyclaceae* and *Comamonadaceae* are known to be facultative anaerobic short-chain fatty acid-utilising denitrifiers (Ginige et al. 2005; Nielsen et al. 2006; Thomsen et al. 2007). Nevertheless, both *Comamonadaceae* and *Rhodocyclaceae* have been previously observed in MFCs. *Comamonadaceae* have been found to dominate the bacterial community in the anode of a cellulose-fed MFC (Rismani-Yazdi et al. 2007). *Rhodocyclaceae* have been found to dominate the bacterial community in the anode of an acetate-fed MFC with an open air cathode (Borole et al. 2009). Furthermore, *Comamonas denitrificans* and *R. ferrireducens* species belonging to the *Comamonadaceae* family are able to generate electricity in an MFC in the absence of oxygen and nitrate (Xing et al. 2008, 2010).

In our PMFC, both oxygen and nitrate were probably present due to the diffusion of oxygen through the roots and the addition of nitrate through the medium. The nitrate flux into the anode was equivalent to 140 g N m^−2^ day^−1^ (2 × 10^−3^ mol_nitrate_ day^−1^, assuming that there was no medium overflow), which is about 125 times higher than the maximum reported rate of nitrogen uptake by emergent macrophytes in a constructed wetland containing *G. maxima* (Tanner 1996). Therefore, a large portion of the added nitrate was denitrified by facultative anaerobic activity as long as short-chain fatty acids were available as electron donors. Short-chain fatty acids were probably generated by the aerobic cellulolytic activity of *Oxalobacteraceae* (Lynd et al. 2002) and the anaerobic cellulolytic activity of *Rhodocyclaceae* (Schellenberger et al. 2010). Offre et al. (2007) associated the presence of *Oxalobacteraceae* and *Comamonadaceae* with mycorrhizal roots and suggested a possible symbiosis between both bacterial families and fungi. The presence of fungi in the outer cortex of the roots in the low-current PMFC was consistent with these findings.

The presence of *Rhodocyclaceae* and *Comamonadaceae* probably had a negative effect on current generation, as these families do not contribute to electricity generation in the presence of oxygen and nitrate. Furthermore, these families consume short-chain fatty acids, which decreases the amount of short chain fatty acids available for current generation and thus negatively affect current generation. The difference in current generation between the high- and low-current-generating PMFC could likely be attributed to difference in presence of *Rhodocyclaceae* and *Comamonandaceae* in the bottom part of both PMFC. In the bottom part of the high-current PMFC, *Rhodocyclaceae* and *Comamonandaceae* were almost absent while they were more abundant in the bottom part of the low-current PMFC. As these families consume short-chain fatty acids, it is likely that their presence resulted in lower current generation because less substrate is available for EAB.

The absence of fungi in the outer cortex of roots in the bottom section of the high-current PMFC, together with possible nitrate depletion may have allowed strictly anaerobic cellulolytic microorganisms (*Clostridiaceae* and *Ruminococcaceae*) and EAB, such as *G. sulfurreducens* and *G. metallireducens*, to proliferate.

### Methanogenesis plays a minor role in competition for electron donor

Methanogens can compete with *G. sulfurreducens* and *G. metallireducens* for electron donors, as they are able to use acetate, formate, methanol, methylamine, H_2_ and CO_2_ (Ishii et al. 2008; Thauer et al. 2008; Thomsen et al. 2007). Quantitative real-time PCR amplification with bacterial and archaeal primers using the same DNA template indicated that considerably less of the DNA present in the sample was derived from archaea than from bacteria. The higher abundance of bacterial DNA compared to archaeal DNA was probably due to the presence of alternative acceptors in the medium, such as oxygen, nitrate and sulphate. As long as these acceptors are present, methanogenic archaea are outcompeted by aerobic bacteria, denitrifiers and/or sulphate-reducing bacteria (Scheid et al. 2004; Thauer et al. 2008). Timmers et al. (2011) did not measure acetate concentrations above 2 mg l^−1^ (the limit of detection) in the PMFCs of which the biological community was analysed. This finding indicated that hydrolysis is the rate-limiting step in that type of cell, assuming that electricity in the PMFC is generated via fermentation of hydrolyses products to acetate and the subsequent oxidation of acetate by *G*. *sulfurreducens* and *G. metallireducens*. The majority of the methanogenic archaeal community, *Methanobacteriacea*, are unable to utilise acetate (Thauer et al. 2008). And therefore do not compete with *G*. *sulfurreducens* and *G. metallireducens* for acetate. This restriction is supported by the finding that of all the archaeal genera found in both PMFCs, only the *Methanosarcina* genera belong to acetate-utilising methanogens (Thauer et al. 2008). *Methanosarcina* comprised, on average, less than 10% of the archaeal DNA in the low-current PMFC and were barely observed in the high-current PMFC. The relative higher abundance of *Methanosarcina* in the low-current PMFC compared to the high-current PMFC likely contributed to competition for acetate. The competition for acetate between *G*. *sulfurreducens* and *G. metallireducens* and *Methanosarcina* likely contributed to the difference in current generation, however less than *Rhodocyclaceae* and *Comamonandaceae*. Furthermore, *Methanosarcina* is a methanogen with cytochrome, which generally have a threshold partial hydrogen pressure above 10 Pa. This consideration may indicate that products other than acetate were formed by fermentation, leading to less acetate production and thus likely a decrease in availability of acetate for generation of current. In addition, the overall abundance of archaeal 16S rRNA genes was considerably lower in the high-current PMFC than in the low-current PMFC. This result leads to the conclusion that the methanogens likely did not consume a major portion of the acetate available for *G*. *sulfurreducens* and *G. metallireducens*.

De Schamphelaire et al. (2010) performed a phylogenetic community analysis on archaea and found that 45% of the archaeal clone sequence was not related to any of the known methanogenic lineages. Of the archaeal clone sequences related to methanogenic lineages, 20% belonged to *Methanobacteriaceae*, 18% to *Methanosarcina* and 10% to *Methanosaetaceae.* In this study, the amounts of the acetate-utilising methanogens *Methanosarcina* and *Methanosaetaceae* were considerably lower (an average of <10% versus an average of 28%). One possible explanation for this discrepancy is the difference in the availability of graphite as an electron acceptor. De Schamphelaire et al. (2010) used a graphite felt in a sediment as the anode electrode for the PMFC, whereas in our setups we used graphite granules (without sediment) as electrodes. The use of graphite granules may have resulted in the increased availability of graphite as an electron acceptor. The limited availability of the graphite felt as an electron acceptor may have given the acetate-utilising methanogens a competitive advantage over EAB. In case the availability of graphite as an electron acceptor indeed limits the competitive advantage of EAB over acetate-utilising methanogens, the availability of graphite as an electron acceptor will be an important factor in the reduction of methanogenesis by the PMFC (De Schamphelaire et al. 2008; Strik et al. 2008).

### Possible electron sinks and electron donor transfer pathways

**Fig. 5:**
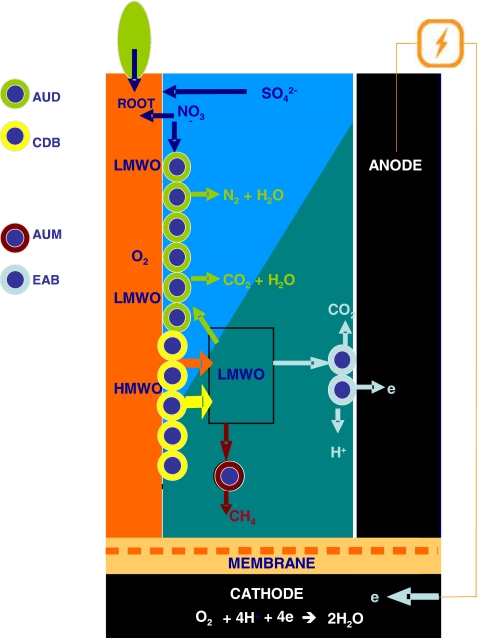
Schematic presentation of the possible oxidation pathways of high molecular weight organic compounds (*HMWO*) and low molecular weight organic compounds (*LMWO*) in the anode of the plant microbial fuel cell (*PMFC*). The *orange arrow* represented the LMWO lost by the plant root; the *yellow arrow* represented the LMWO produced by cellulose-degrading bacteria (*CDB*). The *green*, *light blue* and *brown arrows* represented the possible oxidation pathways and products of the oxidation of LMWO compounds. The *blue colour* represents the volume of the PMFC anode where electrochemically active bacteria (*EAB*) are out-competed by acetate utilising denitrifiers (*AUD*) for LMWO. The *green* colour represents the volume of the PMFC anode where EAB are present and thus compete for LMWO with acetate-utilising methanogens (*AUM*)

Five terminal electron acceptors were present at the anodes of the PMFCs: graphite granules, oxygen, nitrate, sulphate and carbon dioxide. Of these, the latter four are possible electron sinks that do not contribute to current generation. The microbial community at the anode of the PMFC did not contain a large population of sulphate-reducing bacteria, indicating that they were out-competed for electron donor by the facultative anaerobic denitrifiers (such as *Rhodocyclaceae* and *Comamonadaceae*) and EAB (such as *G. sulfurreducens* and *G. metallireducens*) that were present in the anode of the PMFC. This finding is consistent with observations from natural freshwater sediments and wastewater treatment facilities, where the terminal electron acceptors are used in the following order of preference: O_2_, NO_3_^−^, Mn(IV) oxides, Fe(III) oxides, SO_4_^2−^ and CO_2_. Most EAB, such as *G. sulfurreducens* and *G. metallireducens*, reduce Fe(III) oxides in natural sediments (Holmes et al. 2004). Iron reducers have a higher rate of conversion of acetate and hydrogen than sulphate-reducing bacteria and methanogens, which gives them a competitive advantage (Van Bodegom and Scholten 2001). Despite the competitive advantage of EAB, however, acetate-utilising methanogens (*Methanosarcina*) were found. The presence of acetate-utilising methanogens might be explained by the availability of CO_2_ at the root surface, as it is released by the plant roots as the result of metabolic processes. In contrast to CO_2_, graphite granules have limited availability at the root surface due to the porous structure of the anode, which means that not all root surface was in direct contact with graphite granules. Figure 5 shows a schematic presentation of electron donor pathways and electron sinks.

Anaerobic cellulolytic bacteria dominated the area where most of the EAB were found, and more EAB were found on the graphite granules than on the root surface. This result implies that current was generated via the hydrolysis of cellulose and not directly from low molecular weight rhizodeposits. When optimising electricity generation in a PMFC, therefore, the focus should be on root biomass production and effective hydrolysis of the biomass.
